# Hybrid image sensor of small molecule organic photodiode on CMOS – Integration and characterization

**DOI:** 10.1038/s41598-020-64565-5

**Published:** 2020-05-05

**Authors:** Himanshu Shekhar, Amos Fenigstein, Tomer Leitner, Becky Lavi, Dmitry Veinger, Nir Tessler

**Affiliations:** 1 Microelectronics and Nanoelectronics Centers, Electrical Engineering Department, Technion Israel Institute of Technology, Haifa, 32000 Israel; 2 TowerJazz, Tower Semiconductor Ltd., Migdal Haemek, 2310502 Israel

**Keywords:** Electrical and electronic engineering, Optics and photonics

## Abstract

Organic photodiodes (OPDs) for its interesting optoelectronic properties has the potential to be utilized with complementary metal-oxide-semiconductor (CMOS) circuit for imaging, automotive, and security based applications. To achieve such a hybrid device as an image sensor, it is imperative that the quality of the OPD remains high on the CMOS substrate and that it has a well-connected optoelectronic interface with the underneath readout integrated circuit (ROIC) for efficient photogeneration and signal readout. Here, we demonstrate seamless integration of a thermally deposited visible light sensitive small molecule OPD on a standard commercial CMOS substrate using optimized doped PCBM buffer layer. Under a standard power supply voltage of 3 V, this hybrid device shows an excellent photolinearity in the entire bias regime, a high pixel sensitivity of 2 V/Lux.sec, a dynamic range (DR) of 71 dB, and a low dark leakage current density of 1 nA/cm^2^. Moreover, the integrated OPD has a minimum bandwidth of 400 kHz. The photoresponse nonuniformity being only 1.7%, achieved under research lab conditions, strengthens the notion that this fully-CMOS compatible technology has the potential to be applied in high-performance large-scale imaging array.

## Introduction

Organic photodiode (OPD) integrated complementary metal-oxide-semiconductor (CMOS) devices have attracted a great deal of attention because of its potential application in photodetection and imaging technologies^1–5^. Tunable photophysical properties, cheap and straightforward processing methods, ever improving device performance, OPDs have all necessary ingredients to be used in high-end applications such as imaging and video photography. Currently, silicon PD based CMOS image sensor is the leading technology for imaging in the visible spectrum. However, increasing the number of pixels (for higher resolution) and packed with more on-pixel circuitry to meet the market demand, the photosensitivity of the active pixel sensor (APS) based CMOS imager has decreased due to the reduced photosensing area per pixel^6^. Other issues, for example, optical/geometrical cross talk, in standard several µm thick photodiode thickness, degrades the imager resolution and image quality^7,8^. To counter this, several approaches such as incorporating focusing microlens arrays^9^, design of back-side illuminated imager have been adopted to increase the optical fill factor at the expense of increased cost^10^.

Recent developments in the performance of OPDs^1,11–19^ have made them an exciting candidate to employ them as a photosensing element in conjugation with CMOS readout integrated circuit (ROIC) to realize a hybrid imager. Monolithic integration of an OPD on CMOS offers several advantages. First, it can potentially reach a fill factor of 100% as the OPD is directly overlaid in a monolithic fashion on top of a CMOS readout circuit. Second, the absorption spectra can be tuned or broadened by either changing photoactive material or mixing more than one material in the photoactive layer. Third, due to the thin photoactive layer (~100 nm) and absence of metal shields the aperture can be larger than the conventional CMOS imager^5^ which means more photons will be able to reach the photoactive layer making the device more sensitive to light. Lastly, the CMOS provides an efficient read out circuitry which is essential for high quality imaging. Majority of thin film OPDs have been demonstrated using polymers and small molecules as photoactive material. In small molecule based OPDs, organic films are thermally evaporated which can be uniformly and precisely deposited on any size and shape of the substrate.

Small molecules are well equipped for the fabrication of multilayer stacked OPDs where films can be thermally deposited on top of one another without affecting the underneath layer. This is vital as most of the OPDs are stacks of several layers such as photoactive layer, hole blocking layer, and electron blocking layer which play important role in the overall device performance. Although there exist few examples of near-infrared (NIR) sensitive small molecule OPD^15,20^, Vis sensitive small molecules can also potentially be deposited on top of a solution processed NIR sensitive material to envisage Vis to NIR sensitive OPD.

Proof-of-concept hybrid CMOS imagers based on polymeric^21^, colloidal inorganic nanocrystal^22^, and perovskite materials^23^ have been demonstrated. Lim *et al*. used small molecule OPD on a particular indium tin oxide (ITO) designed CMOS circuit^24^. In this work, we present a detail description of design, fabrication, and full characterization of a hybrid device consisting of a visible photosensitive small molecule OPD on a standard commercial CMOS ROIC. Under a standard power supply, the device shows excellent photolinearity, high pixel sensitivity, and low leakage current. The integrated OPD is suitable for high-speed imaging/scanning applications. We believe that an optimized ROIC design, such as 4 T pixel based read out circuit and smaller pixel pitch will improve the results even further. Replacing or adding different photoactive material will be required to broaden the spectral response of the device.

## Results

### Materials and device structure

**Figure 1:**
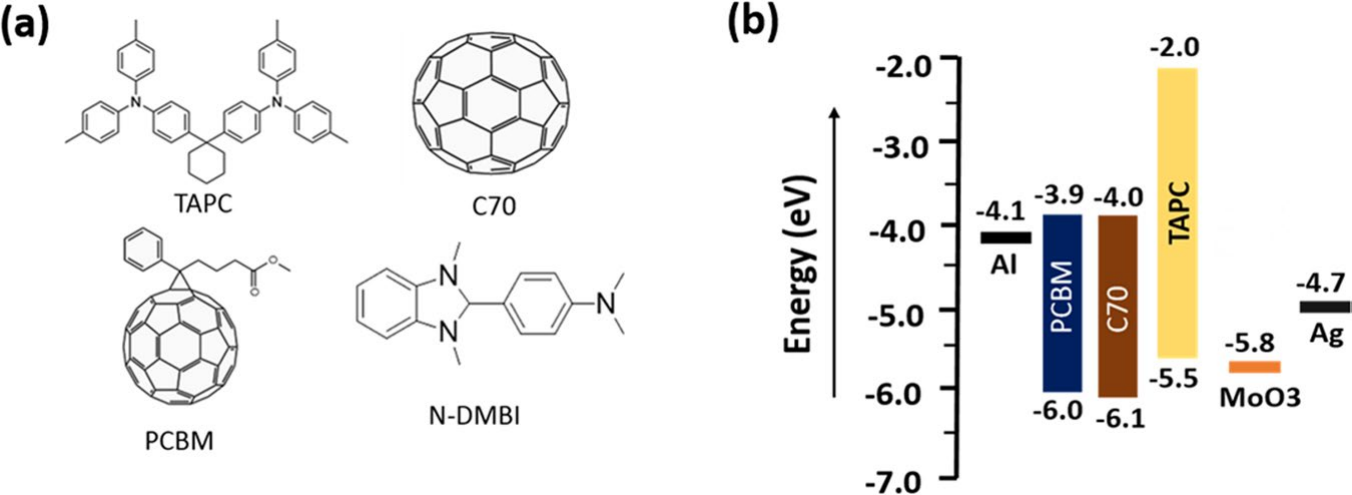
Organic semiconductor materials. (**a**) Molecular structures of TAPC (hole transport material), C70 (electron acceptor and photoactive material), PCBM (electron transport material), and N-DMBI (n-dopant). Films of small molecule TAPC and C70 were thermally deposited while PCBM film was deposited from the solution by spin coating method. (**b**) Energy levels of all the materials used in this work^25–27^.

**Figure 2:**
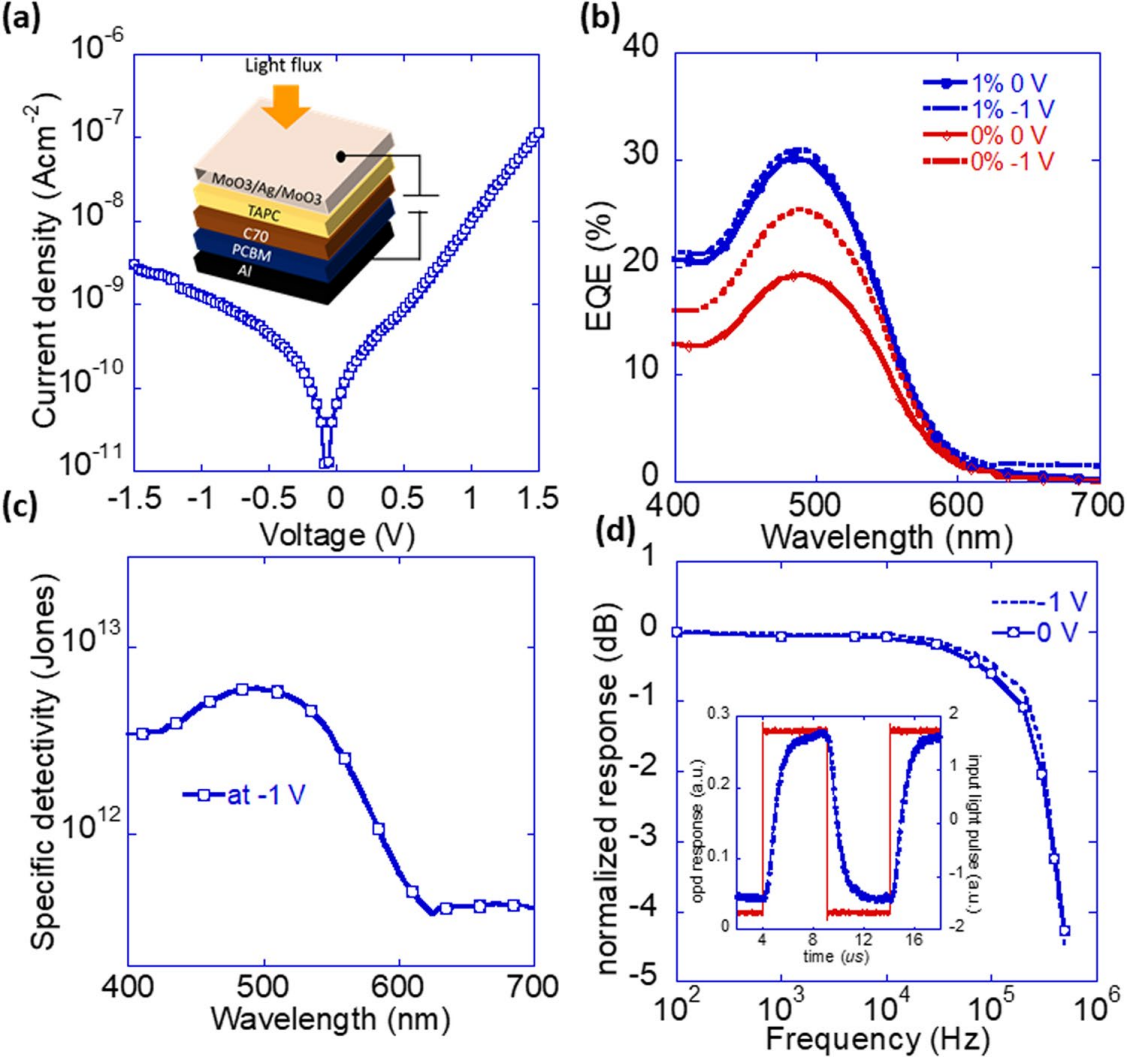
Properties of an inverted device fabricated on an Al test blanket wafer. (**a**) Current density-voltage characteristic of the device measured under dark conditions. The measured dark current density at −1.5 V was 3 × 10^−9^ Acm^−2^. Inset shows the schematic of the top transparent inverted device fabricated on a blanket silicon wafer with Al as a bottom electrode in a stack Si/Al/PCBM (80 nm)/C70 (50 nm)/TAPC (60 nm)/MoO3 (10 nm)/Ag (12 nm)/MoO3 (32 nm). The device area defined by the overlap of the top and bottom electrodes was 18 mm^2^. (**b)** Measured EQE of doped and undoped device under zero and −1 V bias conditions. The doped device shows an EQE of 25% (at −1 V) at a wavelength of 525 nm. (**c**) Calculated specific detectivity at a detection bandwidth of 1 Hz and a bias voltage of −1 V. (**d**) Frequency response of the device at the short circuit and −1 V bias conditions. The −3 dB frequency at zero bias for the device was close to 400 kHz. Inset shows OPD’s output response (blue) as a function of input square pulse light (red) characterized at zero bias.

Molecular structures of all organic materials used in this work are shown in Fig. 1a. All organic materials were used as received. The energy levels of the highest occupied molecular orbital (HOMO) and the lowest unoccupied molecular orbital (LUMO) of materials used in this work are shown in Fig. 1b. Due to a large energy gap of ~3.5 eV, TAPC is largely transparent in the visible spectrum which makes C70 the photoactive material in the device (see Fig. 1, Supplementary Information). The energy offset (~0.6 eV for holes) at the C70/TAPC heterointerface helps dissociation of excitons into free charge carriers. While TAPC forms an heterointerface with C70 and act as a hole acceptor, its high lying LUMO (~2 eV) offers a large electron injection barrier, of ~3.8 eV, at the MoO_3_/TAPC interface. The relatively large barriers at the electrodes (~1.9 eV for holes at Al/PCBM and ~3.8 eV for electrons at MoO_3_/TAPC) are vital in keeping the leakage current low. A cathode buffer layer of PCBM was introduced to mitigate shunts originating from the roughness of the aluminum substrate (see Fig. 2, Supplementary Information) to keep the leakage current as low as possible. Furthermore, the PCBM film was doped (n-doped) to improve the charge transport under low bias conditions so as to ensure linearity even at close to zero bias (see the next section).

### Characteristics of inverted OPD on a test wafer

In order to realize a high-quality OPD on a CMOS readout chip, OPD was first optimized on a test blanket wafer (Aluminum (Al) coated silicon substrate). To keep the electrode property same, Al film on the blanket wafer was deposited under similar conditions as CMOS Al pixel pads. Quality of any OPD is application specific which depends on the working mode of the device. For example, image sensor PDs functions in a charge integration mode which means it is crucial that the leakage current remains as low as possible and device photoresponse characteristics (photo linearity, frequency response, etc.) should ideally be biased independent, down to zero bias. Leakage current is one of the most critical parameters of a photodetector which dictates key photodetector figures of merit namely noise equivalent power (NEP), specific detectivity (D^*^), and linear dynamic range (LDR)^28^. We have shown that the leakage current in our bilayer devices is dictated by the donor/acceptor junction and is correlated with the density of the sub-gap states and the associated recombination-generation processes at that interface^26^. Namely, once reverse injection and extrinsic shunts are minimized, the leakage is governed by the generation-recombination at the junction. Hence, adopting a bilayer structure offered much lower leakage current compared to a device with bulk heterojunction (BHJ) active layer of similar thickness^29^. The significantly higher junction area that supports higher external quantum efficiency (EQE) also results in higher generation-recombination leakage current. Thus, the BHJ device of a similar active layer thickness was inferior to the bilayer as a photodetector due to much higher leakage current.

The bilayer OPD was optimized for the leakage current, low bias photoresponse, and frequency response. An optimized device consisting of aluminum as bottom electrode (cathode), an 80 nm thick n-doped (1%) cathode interfacial layer of PCBM, a 50 nm thick C_70_ as electron acceptor, a 60 nm thick TAPC as hole acceptor followed by a transparent anode consisting of MoO_3_ (10 nm)/Ag (12 nm)/ MoO_3_ (32 nm) was fabricated^29^. A schematic sketch of the complete device is displayed in the inset of Fig. 2a (for detail see the Methods section). We have demonstrated that the incorporation of PCBM interlayer improves the leakage current by reducing the effect of the substrate roughness. By doping the PCBM film, the electrical contact between electrode/PCBM and transport of photogenerated carriers across the PCBM can be improved^29^. The measured dark current density-voltage response of the device is shown in Fig. 2a. The device exhibited a leakage current of ~3 nAcm^−2^ at − 1.5 V and ~1 nAcm^−2^ at − 1 V reverse bias conditions. Plots of the measured spectral EQE of the device under a bias of 0 V and −1 V are shown in Fig. 2b. The device shows an EQE of ~30% and ~25% measured at 500 nm and 525 nm wavelengths of light respectively at short circuit condition. Furthermore, the device shows a bias independent EQE which means photogenerated charges are effectively transported within the device and collected at the respective electrodes. To demonstrate the advantage of doping the PCBM layer, Fig. 2b is also shows the EQE of an undoped device (i.e., 0% doped PCBM). Compared to the doped device, the undoped device shows a lower EQE of ~19% and ~16% at 500 nm and 525 nm respectively at short circuit condition. The EQE of the undoped device has a bias dependency which increased from 19% (V = 0) to 25% (V = −1 V) at a wavelength of light of 500 nm.

Figure 2c shows the calculated spectral specific detectivity of the device for an electrical bandwidth of 1 Hz and a bias of −1 V (for detail see the Supplementary Information). At 500 nm light wavelength and under a bias of −1 V, the calculated specific detectivity of 1% doped devices was ~6 × 10^12^ cm Hz^1/2^ W^−1^ (Jones)^29^. We note that in the detectivity calculation, flicker and generation-recombination noise components were not taken into account^30^. We also note that the frequency dependence noises such as flicker noise are dominant at frequencies much below the operating frequency of imaging cameras^31^.

Finally, the frequency response of the device was characterized under a modulated input optical signal (for detail see the Methods section). The cut-off frequency (bandwidth) was determined by the frequency of input light modulation at which the photodiode output response is −3 dB lower than the low-frequency signal. The cut-off frequency of the device measured at 0 V and −1 V bias are shown in Fig. 2d. The device shows a bias independent frequency response whose −3 dB frequency was close to 400 kHz. This high bandwidth achieved is more than adequate for imaging applications such as required in digital still cameras and video cameras. An alternative method to characterize the photodiode’s speed is to quantify its rise or fall times. The rise time is defined as the time it takes the output signal to increase from 10% to 90% of the final output level^29^. In case the bandwidth is limited by RC time constant (*f*_*_RC*_), the rise time (*t*_*r*_) can be expressed as^28,32^

1 $${t}_{r}=\frac{0.35}{{f}_{\_RC}}$$

Using the −3 dB frequency of 400 kHz in Eq. (1) the calculated *t*_*r*_ was~0.9 µs. The measured rise time of the device was about 1.2 µs (see inset of Fig. 2d) which is close to the −3 dB frequency corresponding rise time. From this result, it appears that the cut-off frequency of our device is determined by the characteristic RC time. The bandwidth can be further improved by reducing the capacitance of the RC time, for example, by shrinking the device area of 18mm^2^ until it is limited by the carrier transit time (t_tr_)^30^.

### Integration of OPD on CMOS readout circuit and its characterization

**Figure 3:**
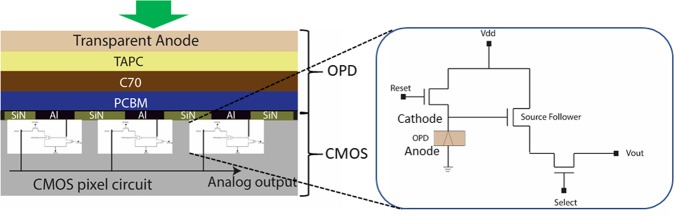
Hybrid device (imager) architecture. Schematic of the investigated device where OPD is stacked on the top of a CMOS substrate. The bottom electrode of the OPD is defined by the CMOS aluminum pad (80 µm × 80 µm) resulting in an active device area of 0.0064 mm^2^. Right: The schematic represents the image sensor’s pixel circuit based on 3 T active pixel architecture. The reset transistor is used to reset the OPD to a starting point voltage V_dd_ – V_t reset_. At the end of the exposure period, the photogenerated voltage is buffered by the source follower transistor. For read-out and control, pixel is selected by a select transistor.

To realize a hybrid imager, the optimized OPD was fabricated on top of a CMOS substrate hosting the readout circuit (see the Methods section). The layer structure diagram showing different stacks of the OPD integrated CMOS chip is presented in Fig. 3. The uppermost metal layer (Al; shown in the black color in Fig. 3) of the CMOS served as the bottom electrode (cathode) for the OPD. The pixel electrodes are separated by insulating passivation layer (SiN; shown in the light green color in Fig. 3). The hybrid imager pixel based on 3 T active pixel architecture, consisting of an OPD, a reset transistor, a source follower transistor, and a select transistor^33^, is shown in the enlarged diagram in Fig. 3. For pixel characterization, an external voltage bias was provided to Al pixel electrodes via a reset transistor. The Al electrode also connected the OPD with the readout circuit of the pixel (via Source Follower) to collect the photoelectrical signal. The signal read-out mechanism is explained in the Methods section.

**Figure 4:**
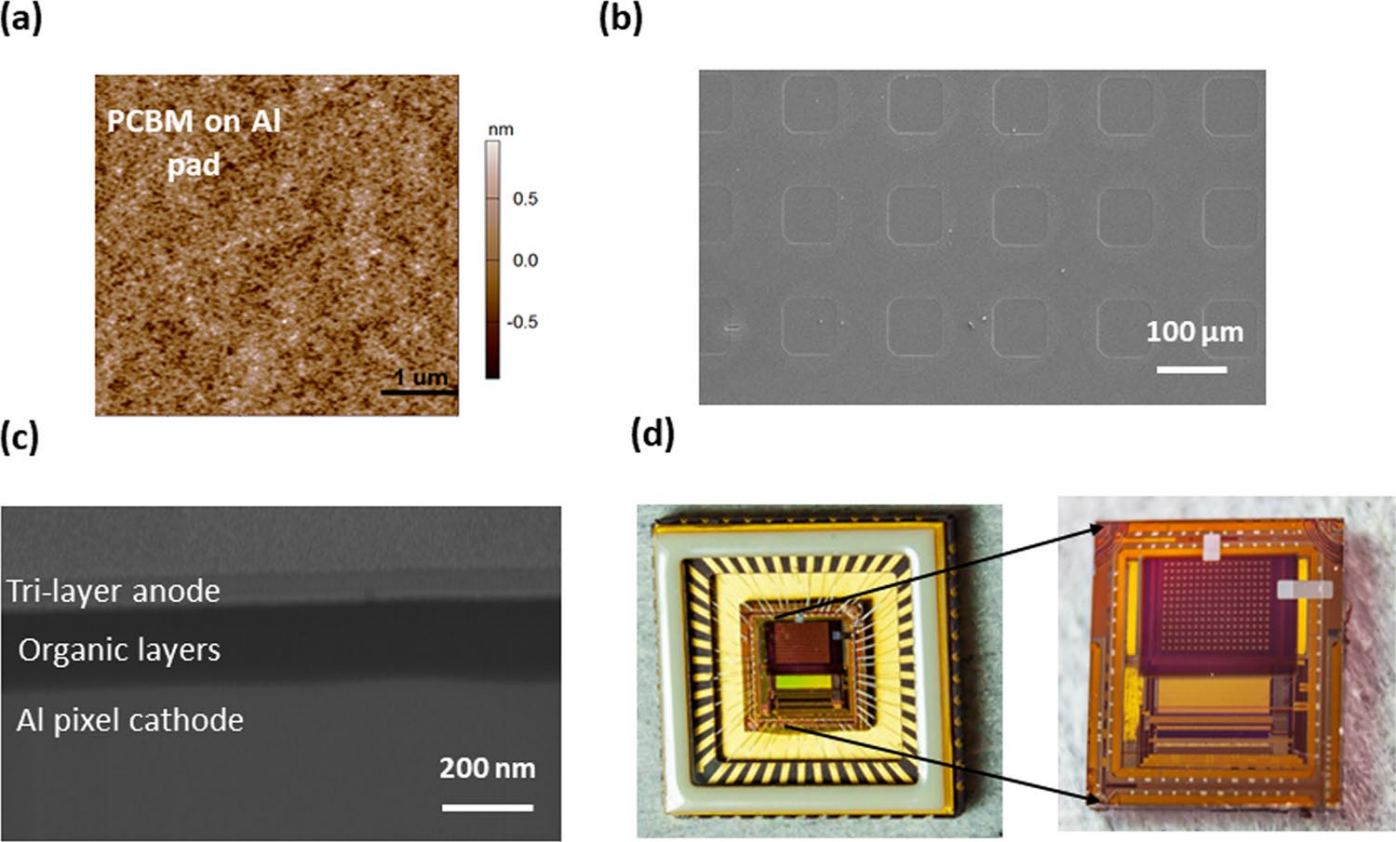
Physical characterization of pixels. (**a**) Tapping mode AFM scan of the PCBM coated aluminum pixel pad highlighting the surface roughness. The rms surface roughness was in the range of ~1 nm. The scanned surface area is 5 µm x 5 µm. (**b**) Top view SEM micrograph of OPD integrated pixel pads. (**c**) Cross-sectional SEM image of a pixel showing the uniformity of different stacking layers. Unable to differentiate between different organic layers the SEM image shows PCBM, C70, and TAPC as a single layer of thickness ~200 nm. (**d**) Photograph of the fabricated device bonded to a chip carrier. Right: Same device (outside chip carrier) showing pixelated area with OPD on it. Ag electrodes (in white) connecting the top electrode to the ground of the CMOS chip.

Figure 4 shows the physical characteristics of the pixel pads at the various stages of the OPD integration process. Figure 4a shows the AFM scan of the doped-PCBM coated Al pixel pad. PCBM coating of the pixel pad resulted in a smooth surface with root mean square (rms) roughness of ~1 nm. Figure 4b shows top view SEM micrograph of a section of the pixelated area of the CMOS substrate at the end of the OPD deposition process. Shown in Fig. 4c is a cross-sectional view of the pixel highlighting the homogeneity/uniformity of the multilayer stack within the pixel. Figure 4d shows the photographs of the hybrid device and same device bonded on a chip carrier.

As the limiting factor for such future hybrid image sensors may be the integration of the OPD with the CMOS substrate, we present here a detailed pixel-level analysis of such hybrid sensor. To the best of our knowledge, this is the first example of detail integration and full pixel characterization of thermally deposited small molecule based OPD on a standard CMOS substrate. In order to evaluate the performance of the hybrid pixels, the fabricated device (shown in Fig. 4d) was characterized as an image sensor at Tower’s characterization lab. All measurements were done in an ambient condition at room temperature. We note that, as the organic layers were evaporated through a shadow mask of opening equivalent to the size of the pixelated area of the ROIC, due to the offsetting in alignment, pixels on the outer edges showed large photoresponse nonuniformity. In total, approximately 20 pixels (out of 221) were excluded from the analysis. Such nonuniformities, however, can be mitigated by optimizing the fabrication process. We mention that due to the limited number and relatively large size of pixels no attempt was made to capture any image using our hybrid device and the focus was given to pixel specific characterizations.

### Pixel transfer curve and sensitivity

**Figure 5:**
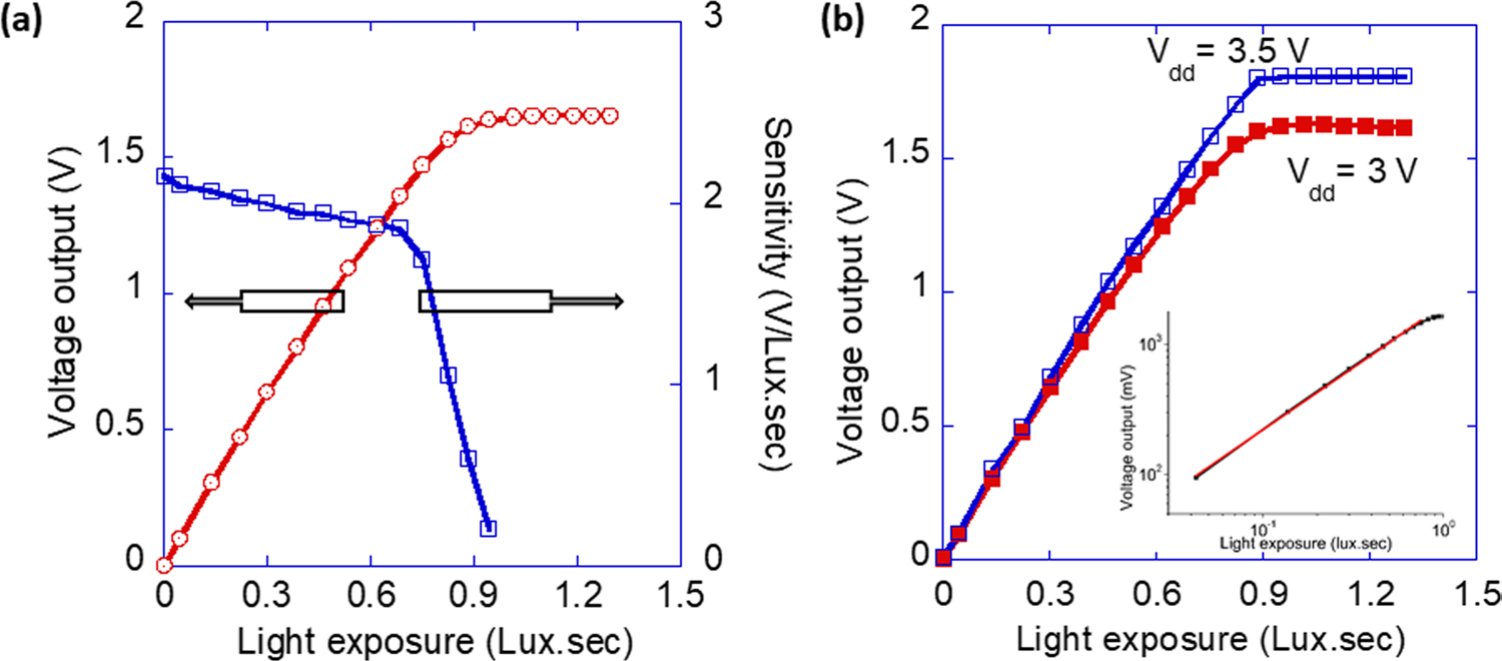
Pixel output characteristics. (**a**) Average pixel output response (red line) at V_dd_ = 3 V as a function of light exposure illuminated using a green light emitting diode of wavelength 523 nm. Pixel characterization was done under different light exposure by varying the light intensity from 5.36 ×10^−7^ mWcm^−2^ to 6.48 ×10^−3^ mWcm^−2^ at a 200 ms integration time. Pixel sensitivity (V/Lux.sec) (blue line) was extracted from the slope of the pixel output response. (**b**) Pixel output response at V_dd_ = 3 V and 3.5 V. Increasing the V_dd_ from 3 V to 3.5 V resulted in a higher saturation voltage of 1.8 V compared to 1.6 V (at V_dd_ = 3 V) and a slightly improved sensitivity 1.98 V/Lux.sec (at V_dd_ = 3 V) to 2.12 V/Lux.sec (at V_dd_ = 3.5 V). The inset highlights the linearity of the output response at V_dd_ = 3 V plotted on a log-log scale.

For this measurement the light intensity was increased in steps from 5.36 × 10^−7^ mWcm^−2^ to 6.48 × 10^−3^ mWcm^−2^ at a fixed integration time (t_int_). To determine pixel’s full well capacity (FWC)/saturation voltage and characterize its behavior at low reverse bias conditions (<−1 V), t_int_ was set to 200 ms. Figure 5 depicts the device output response (average output response of all pixels) under increasing light exposure. Output response and sensitivity of the device at a shorter integration time (t_int_ = 20 ms) adequate for video photography was also recorded (see Fig. 4 in the Supplementary Information). Figure 5a (left y-axis) shows the average output response, also known as pixel transfer curve, of the device characterized at a standard CMOS power supply voltage of V_dd_ = 3 V.

Pixel output voltage shows a linear response in the entire exposure regime up to the point where the output voltage saturates. Under this measurement condition, the pixel has a saturation voltage of~1.6 V. The highest output voltage which deviates by less than 5% from the linear response was found to be 1.46 V at an exposure of 0.75 Lux.sec. Sensitivity of the pixel is determined by taking the derivative of the transfer curve (see Fig. 5a (right y-axis)). The device shows a relatively uniform sensitivity of 1.98 V/Lux.sec in the linear regime of the transfer curve. This value is much higher than reported by Lim *et al*. for a hybrid color imager on a newly designed CMOS circuit^24^. Figure 5b shows the pixel transfer curve under different bias conditions. This means OPD is differently biased and the starting voltage at the output node of the OPD is also different. Increasing the V_dd_ to 3.5 V resulted in a higher saturation voltage of 1.8 V compared to 1.6 V at V_dd_ = 3 V. In the case of V_dd_ = 3.5 V there is a sharp onset of saturation which is a result of the maximum output voltage of the pixel being limited by the circuitry of the pixel itself and not by the OPD. There is a slight improvement in the pixel sensitivity with increasing bias (1.98 V/Lux.sec (at V_dd_ = 3 V) to 2.12 V/Lux.sec (at V_dd_ = 3.5 V)). This improvement is most likely due to electric field enhanced charge transport and collection efficiency leading to reduction of charge recombination losses within the OPD.

### Pixel conversion gain and capacitance

**Figure 6:**
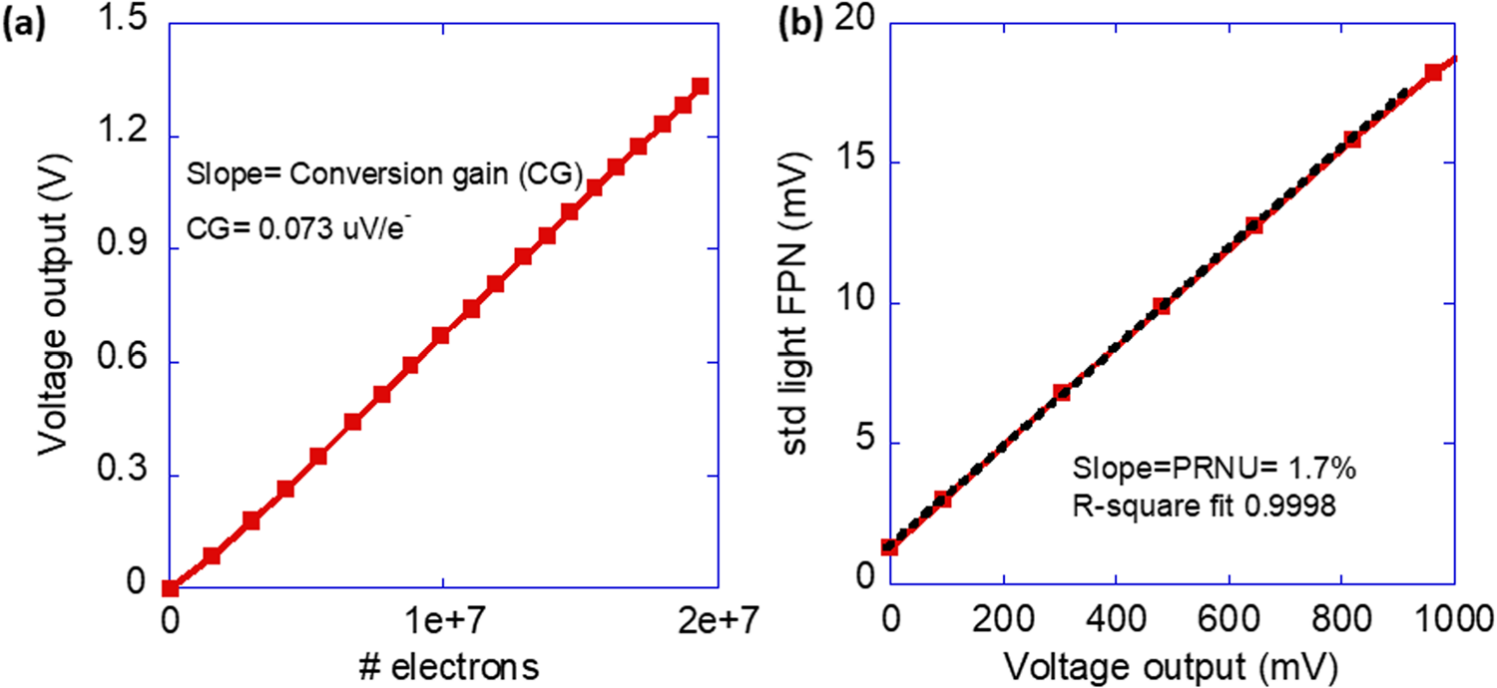
Pixel conversion gain and photoresponse uniformity. (**a**) Conversion gain (V/e^−^) of the pixel extracted from the photoresponse curve. The number of photogenerated electrons was calculated from a known EQE measured from an inverted OPD fabricated on test wafer (Fig. 2b) and the average number of photons impinging on the pixel. The extracted conversion gain for pixel (pad size 80 µm) was 0.073 µV/e^−^. (**b**) Measurement of standard deviation (std) of the output voltage signal (Fixed pattern noise (FPN)) under different light exposure as a function of the mean output signal. The slope of the linear part of the graph known as PRNU or gain FPN was 1.7% of the output signal.

A key parameter of an image sensor pixel is conversion gain (CG) or conversion factor^34^. It measures the change in the output response (here voltage) per electron generated by the pixel and is represented in (V/e^-^). Figure 6a shows the pixel output voltage as a function of the photogenerated electrons collected at the output node of the OPD (i.e. averaged over 200 pixels). From the slope of the curve, the calculated conversion gain of the pixel is 0.073 µV/e^-^ (for details see the Supplementary Information). In short, for the 3 T APS ROIC the conversion gain is inversely proportional to the pixel’s capacitance. The relatively low value reported above is associated with the large pixel size of 80 µm x 80 µm. To verify it is the PD capacitance that determines the conversion gain we estimate the capacitance of the charge storage node. The charge to voltage conversion can be expressed as

2 $$\Delta {V}_{out}=\frac{\Delta Q}{{C}_{OPD}}$$

where $\Delta {V}_{out}$ is the voltage corresponding to the photogenerated charge $\Delta Q$ and ${C}_{OPD}$ is the capacitance of the photodiode. The capacitance of the photodiode can be approximated from the below expression^7^

3 $${C}_{OPD}=\frac{{A}_{SF}\times q}{CG}$$

where ${A}_{SF}$ is the source follower gain, $q$ is the elementary charge, and rest of the terms have their meaning as mentioned above. In our case, ${A}_{SF}$ is ~0.9 and the conversion gain is 0.073 µV/e^−^. The pixel capacitance calculated from Eq. (3) was found to be ~1.98 pF. Alternatively, the capacitance of our photodiode can be estimated from a parallel plate capacitor model using the below expression

4 $${C}_{OPD}=\frac{{\varepsilon }_{0}\times {\varepsilon }_{r}\times A}{d}$$

where ${\varepsilon }_{0}$ is the vacuum permittivity, ${\varepsilon }_{r}$ is the relative permittivity of organic layers, *A* is the area of the capacitor, and *d* is the thickness of the organic layer. Using values ${\varepsilon }_{0}$= 8.85 ×10^−12^ Fm^−1^, ${\varepsilon }_{r}$= 4, *A* = 80 µm x 80 µm, *d* = 100 nm the calculated capacitance using Eq. (4) is ~2 pF. We note that in this calculation the thickness of the doped PCBM layer has been excluded. The capacitance values obtained using Eqs. (3) and (4) are in good correlation which also verifies the value of the conversion gain obtained from the photoresponse curve (see Fig. 6a). From Eq. (2) it is clear that a large capacitance will result in a small change in the voltage. One way to increase the conversion gain is by reducing the size of the pixel. However, this will also limit the well capacity of photodiode at a given V_dd_ which will limit the dynamic range.

### Photo response uniformity of the hybrid device

Ideally, the pixel output voltage under a given measurement condition should be uniform across the pixel array. However, various factors such as variation in the threshold voltage (V_tn_) of pixel level reset transistors, the difference in the photoresponse of OPDs due to any nonuniformity among them, and other spatial nonuniformity lead to a spatial noise also known as fixed pattern noise (FPN)^7^. Fig. 6b depicts the FPN of the imager as a function of the output voltage under the light. FPN is calculated as the standard deviation of the pixels’ output under uniform illumination. From the response (Fig. 6b) we can see that the FPN increases linearly with the output voltage. The slope of the linear part of the curve also called photoresponse nonuniformity (PRNU) or gain FPN^35^ that describes how fast FPN increases with the exposure is expressed as

5 $${\rm{PRNU}}=\frac{{\rm{FPN}}\,{[{\rm{V}}}_{std}]}{{\rm{Mean}}\,{[{\rm{V}}}_{{\rm{signal}}}]}$$

The extracted PRNU for the device was ~1.7% of the output signal. A large FPN would degrade the signal-to-noise ratio (SNR) which is the ratio between the signal and the total noise. Typically, CMOS image sensors will have their PRNU spec at below 1%^8,36^.

### Dark leakage current of hybrid pixel

**Figure 7:**
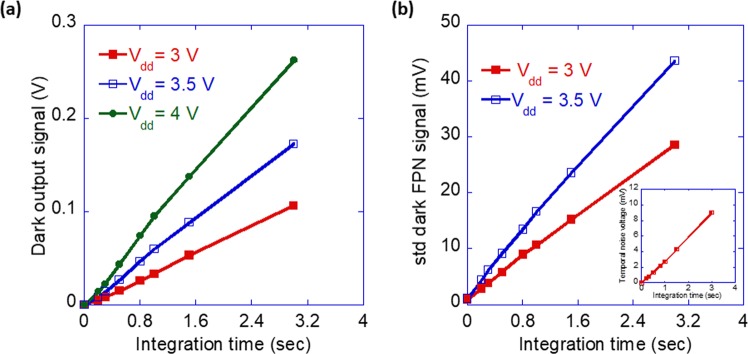
Pixel response in the dark state. (**a**) The average dark output response of pixels measured under different integration times (1 ms to 3 s) at different V_dd_. The calculated average dark current density (A/cm^2^) per pixel at V_dd_ = 3 V, 3.5 V, and 4 V was 1.07, 1.8, and 2.62 nA/cm^2^ respectively (see the Supplementary Information for detail). (**b)** Measured dark FPN of the device as a function of integration time at V_dd_ = 3 V, 3.5 V. The inset shows the temporal noise of pixel at different integration times. In both the figures symbols are measurement points, and solid lines are a guide to the eye.

The output signal of an image sensor under dark condition is unwanted and is considered as one of the main sources of the noise^36,37^. This is because signal present in the dark will also appear under illumination and will convolute the main signal. The dark signal thus lifts the noise floor up, affecting the dynamic range (DR) of the image sensor which defines sensor’s capability to efficiently capture images under bright conditions (sunny day) and weak light conditions (evening twilight, dark cloudy day). A large dark noise signal would reduce the SNR and degrade the image quality^35,38^. To estimate the dark current in our OPDs, the device was characterized in the dark with increasing integration time (from 1 ms to 3 s) at V_dd_ = 3 V, 3.5 V, and 4 V (see the Methods section). Figure 7a shows the average output voltage of the device in dark as a function of the integration time. Assuming the output voltage is due to the dark or leakage current of the OPD, the dark current is integrated as dark charge at the charge storage node of the pixel and is expressed as (e^-^/sec) or current density (A/cm^2^) per pixel. Using the conversion gain of our pixel of 0.073 µV/e^-^, the dark output voltage equivalent charge carrier (electrons) number can be calculated using the below equation

6 $${N}_{dark}[{e}^{-}]=\frac{\Delta {V}_{dark}[\mu V]}{0.073[\mu V/{e}^{-}]}$$

From the number of dark electrons collected at the charge storage node, the absolute dark current of a pixel can be estimated as

7 $${I}_{dark}[A]=\frac{{N}_{dark}[{e}^{-}]\times q}{{t}_{\mathrm{int}}[\text{sec}]}$$

Using Eq. (7) the calculated dark current of our OPD at V_dd_ = 3 V was ~70 fA (or 1.07 nA/cm^2^). As the bias across the OPD is increased the corresponding dark current increased too. At 4 V V_dd_ the dark current was 2.62 nA/cm^2^. The dark current density of our hybrid pixel at 4 V V_dd_ is about an order lower than that of Baierl *et al*. reported for sprayed coated OPD on CMOS^21^. We note that the calculated leakage current density of the hybrid pixel is somewhat lower compared to the measured leakage current of the inverted OPD fabricated on test blanket wafer (see Fig. 2a). This is after accounting for the fact that at any V_dd_ the net bias across the OPD will be affected by the V_tn_ of the reset transistor of the pixel and is approximately given by ~V_dd_ − V_tn_. This reduction in leakage current is probably due the smaller area of the pixel pads compared to the inverted OPD on the test wafer which reduces the contributions of external factors such as particles or random shunts.

### Dark spatial and temporal noise

Noise appearing in the dark output signal in an image sensor is mainly composed of two components, spatial noise due to the nonuniformity in the photodiode dark current and temporal/random noise that is due to the dark shot noise, thermal noise, 1/f noise and other noises appearing from pixel amplifier and read-out circuit, etc^7,35^. The dark signal (dark current) varying from pixel to pixel over the pixel array introduces dark FPN which is measured in a similar manner to the (light) FPN described above. In Fig. 7b the standard deviation of the dark output signal of the device characterized at V_dd_ = 3 V and 3.5 V as a function of integration time is shown. The slope of dark FPN vs integration time curve gives the dark signal nonuniformity (DSNU) of the device which in this case is ~9 mV/sec at V_dd_ = 3 V. Temporal noise in our hybrid device was calculated from the variance of the pixel value over a series of frames taken in the dark. Among temporal noise sources in the dark, shot noise due to photodiode dark current is one of the dominant mechanisms. To estimate where does the dark temporal noise stand in comparison to the dark FPN, dark temporal noise signal consisting shot noise, temporal reset noise, thermal noise etc. of the device at different integration times at an external bias V_dd_ = 3 V is shown in the inset of Fig. 7b. At V_dd_ = 3 V and t_int_ = 20 ms the temporal noise level of ~500 µV was lower than the FPN noise of ~1 mV. These noise values are quite low and are even better than the values reported on OPD based hybrid imager^21^.

### Dynamic range

Lastly, the DR of this hybrid device is estimated using the below expression

8 $$DR[dB]=20\times \,\log \,\frac{{V}_{\max }[mV]}{{\rm{Noise}}\,{\rm{floor}}[mV]}$$

where ${V}_{\max }$ is the maximum output signal under the light that can be achieved for the given pixel and read-out circuitry and was found to be ~1.8 V. Taking a value of 0.5 mV as the signal noise floor (temporal noise at t_int_ = 20 ms), the calculated DR was about 71 dB which is close to state of the art hybrid imagers^5,39^.

## Discussion

The summary of the results of the hybrid device developed in this work is tabulated in Table 1 below. The image sensors were not encapsulated and were kept and measured under ambient conditions at room temperature.

**Table 1:** Performance of the hybrid image sensor.

Parameter	Value
Process	0.18 µm, 1-Poly/4-Metal
Signal readout circuit	3 T APS
Pixel array	17 (w) × 13 (h)
Pixel size	80 µm
Bias (V_dd_)	3 V
Pixel saturation voltage	1.8 V (Limited by ROIC)
Pixel sensitivity	2 V/Lux.sec
Pixel conversion gain	0.073 µV/e^-^
Pixel capacitance	~2 pF
Pixel dark current	~1 nAcm^−2^ (V_dd_ = 3 V)
OPD EQE	25% at 525 nm at V = 0 V, −1 V
PRNU	1.7%
Light input	Green LED 523 nm

Specification of the CMOS substrate and fabricated hybrid imagerl.

The above results show a successful seamless integration of a small molecule OPD on a standard commercial CMOS circuit. The key was the use of moderately doped PCBM layer along with an optimized photodiode layer structure. The use of evaporated small molecules implies that moving from single color to multicolor requires only the shadow mask technology that is well developed for commercial organic light emitting diode (OLED) displays. The device presented above shows excellent photolinearity at low bias conditions which allow utilizing the full well capacity of the OPD. This might be useful in still photography that requires long integration time or imaging in intense illumination conditions. The fabricated device has a low leakage current at the standard operating voltage which highlights the quality and robustness of the OPD. Moreover, the high-frequency response of the OPD ensures normal operation of the device under short integration times (~1 µs). Although due to the bi-layer photoactive film structure the EQE of the device is relatively low compared to a conventional CMOS imager; this can be enhanced by adopting different materials’ (donor/acceptor) combinations. To keep the overall performance level, some care must be taken that the dark response of the device doesn’t degrade. The monolithic integration of OPD allows shrinking the pixel size to much lower values until it is limited by lithography or any CMOS processing step. Our results show that by reducing the pixels’ size and adopting the CDS scheme will result in even higher quality imager.

## Methods

### Organic photodiode (OPD) fabrication on a test wafer

Using lithography and wet etching technique aluminum-coated wafer was patterned into pixelated substrate. Each substrate (size 12 mm × 12 mm) had two spatially and electrically separated electrodes of size 18 mm^2^. To remove any oxide layer from the ambient exposed aluminum pixel pads, the substrates were first cleaned in dilute ammonium hydroxide (NH_4_OH 30%) solution for 40 sec followed by rinsing in isopropanol. Afterward, the substrates were well dried under nitrogen flow and brought in an N_2_-filled glovebox (O_2_ 9ppm. H_2_O < 0.1ppm) for further processing. First, an 80 nm thick layer of 4-(1,3-Dimethyl-2,3-dihydro-1H-benzoimidazol-2-yl)phenyl)dimethylamine (N-DMBI; Sigma Aldrich 99.9%) doped 6,6-phenyl C_61_ butyric acid methyl ester (PCBM; NANO-C 99.5%) was deposited by spin-coating. For this purpose, a 30 mg/ml solution of PCBM dissolved in chlorobenzene (CB) was stirred and heated (at 70^0^ C) for 24 hours; After that the solution was filtered (0.2 μm PTFE) and mixed with N-DMBI solution (in CB) in 1 molar %, and left overnight to mix well before spin coating. The films were spin-coated inside an N_2_-filled glovebox and annealed at 75^0^ C for 30 minutes under nitrogen atmosphere. Directly afterward, through a shadow mask a 50 nm thick film of Fullerene-C70 (C_70_; Lumtec (HPLC)), and a 60 nm thick film of Di-[4-(N,N-di-p-tolyl-amino)-phenyl] cyclohexane (TAPC; Lumtec (HPLC)) were thermally evaporated to serve as acceptor/donor bi-layer. Finally, a transparent tri-layer anode consisting of 10 nm thick film of Molybdenum trioxide (MoO_3_, Sigma 99.99%), 12 nm thick silver (Ag) and a 32 nm thick MoO_3_ was evaporated^29^. All layers above PCBM were deposited without breaking the vacuum. The evaporator base pressure was 4e^−7^ mbar.

### OPD Characterization

Dark current-voltage of OPDs was characterized with a semiconductor parameter analyzer (B1500 A, Agilent Technologies) inside an N_2_-filled glovebox. Spectrally resolved EQE was performed outside the glove box with measured samples kept in nitrogen atmosphere inside a holder. Light from the monochromator (Cornerstone™ 130) was chopped at 80 Hz, and the signal was read using a lock-in amplifier (EG & G 7265). Frequency measurements were conducted using a green light emitting diode (LED) modulated by a square pulse using AFG3252 Tektronix, waveform generator. The dynamic photocurrent response of the OPDs was recorded using a digital oscilloscope (DPO3034 Tektronix)^29^.

### OPD fabrication on CMOS

OPDs were fabricated directly on top of CMOS substrates that contained the read-out circuitry of the image sensor. CMOS substrates were designed and manufactured at the facility of Towerjazz Ltd. Israel. The CMOS substrate was prepared in a 0.18 µm (1 poly, 4 level metal process) standard CMOS technology. The active pixel size, defined by the bottom aluminum pixel pad, was 80 um x 80 um. The pixel area consisted of a total of 221 such pixels arranged in an array of 17 × 13. The CMOS substrates were first cleaned in a solution of diluted NH_4_OH. Afterward, an 80 nm thick doped PCBM was directly spin coated on the CMOS substrate (see Methods section OPD fabrication on test wafer). To remove the PCBM layer from the bonding pins of the chip, PCBM layer was patterned using i-line photolithography with a negative orthogonal photoresist (OSCoR 5001). After lithography, PCBM film from the bonding pin area was removed by oxygen plasma reactive ion etching. Finally, the photoresist covering the pixel area was stripped in a stripper to leave PCBM on the pixel area only. Directly afterward, through a shadow mask a 50 nm thick film of C70 and a 60 nm thick film of TAPC were thermally evaporated to serve as acceptor/donor bi-layer. Finally, a tri-layer anode consisting of 10 nm thick film of MoO_3_, 12 nm thick Ag and a 32 nm thick MoO_3_ was evaporated^29^. To ease the bonding to this thin tri-layer electrode, a 150 nm thick silver using a different shadow mask was evaporated to connect the ground of the CMOS read-out to the tri-layer electrode (see Fig. 4d).

### SEM and AFM characterization

Top and cross-sectional scanning electron microscopy (SEM) images were captured using the focused ion beam (FIB) (Helios NanoLab DualBeam) milling technique. Atomic force microscopy (AFM) scan of the PCBM coated Al pixel pad was acquired using an MFP-3D Infinity AFM operated in the tapping mode^29^.

### Device bonding and encapsulation

To characterize the device as an imager, the CMOS chip was integrated into a chip carrier. The fabricated device was wire bonded to the pins of a chip carrier (14 mm ×14 mm ceramic package for image sensor. see Fig. 4d). Finally, the device was encapsulated with a glass lid (14 mm ×14 mm) in an N_2_-filled glovebox. All encapsulation related parts were provided by TowerJazz, Tower Semiconductor Ltd., Migdal Haemek 2310502, Israel.

### Imager readout circuit architecture

The CMOS chips were provided with the necessary power supply and input signals via an imager test board. The test board hosted key components such as timing and reference signal controller circuit, analog-to-digital converter (ADC), and an interface for transferring the digital data to a computer. The image sensor pixel architecture is based on a standard 3 T (3 transistor) active pixel sensor (APS). The average output signal of a pixel is usually contaminated with temporal as well as fixed pattern noise (FPN) components. To minimize the FPN due to the dispersion of the threshold voltage (V_tn_) of the pixel reset transistors, a non-correlated double sampling (non-CDS) in the column amplifiers was performed before sending the analog output signal to the ADC circuit. Photogenerated charges underneath the pixel circuitry can diffuse to the photodiode node which can potentially add to the spatial noise. This was taken care by forming an n-well around the pixel transistors.

### Imager characterization

Pixels (matrix of 17 (w) x 13 (h)) were characterized by illuminating the sensor with a homogenous light using a green light emitting diode of wavelength 523 nm. Pixel characterization was done at different bias voltages (V_dd_) under various light exposure by varying the light intensity from 5.36 ×10^−7^ mWcm^−2^ to 6.48 ×10^−3^ mWcm^−2^ at a fixed integration time. Pixel output signal under light was also characterized as a function of integration time varying from 10 ms to 200 ms. The longer integration time with 200 ms was used to record the linearity of the pixel photoresponse in the low bias regime (V < −1 V) of the photodiode and to determine the pixel saturation voltage. The pixel sensitivity (V/Lux.sec) was determined from the slope of the average pixel output response. Conversion gain (V/e^-^) of the pixel was extracted from the photoresponse curve with a known quantum efficiency measured from an inverted OPD fabricated on the test wafer (see OPD characterization in the Methods section). The average dark output signal was measured in dark under different integration times (1 ms to 3 s) at specific V_dd_ (as mentioned in the main text). Pixel dark current (e/sec or A/cm^2^) was estimated from the measured average dark signal and the conversion gain. Device photoresponse nonuniformity (PRNU) and dark signal nonuniformity (DSNU) were estimated from the calculated standard deviation of the respective output signals. Pixel dark temporal noise was calculated from the signal variance. The dynamic range of the pixel was estimated from the maximum output voltage (saturation voltage) and the temporal noise floor of the pixel.

## Supplementary information

Supplementary Information.
