# Supplementary material for: Hybrid image sensor of small molecule organic photodiode on CMOS – Integration and characterization
Source: Sci Rep. 2020 May 5;10:7594. doi: 10.1038/s41598-020-64565-5 (PMC7200686; doi:10.1038/s41598-020-64565-5)
Supplement: Supplementary file 1 — Supplementary Information. [file 41598_2020_64565_MOESM1_ESM.docx]

**Supplementary Information**

Hybrid image sensor of small molecule organic photodiode on CMOS – Integration and characterization

Himanshu Shekhar,^1^ Amos Fenigstein,^2^ Tomer Leitner ^2^ Becky Lavi ^2^ Dmitry Veinger ^2^ and Nir Tessler ^1*^

^1^ Microelectronics and Nanoelectronics Centers, Electrical Engineering Department, Technion Israel Institute of Technology, Haifa 32000, Israel

^2^ TowerJazz, Tower Semiconductor Ltd., Migdal Haemek 2310502, Israel

^*^ E-mail: [nir@technion.ac.il](mailto:nir@technion.ac.il)


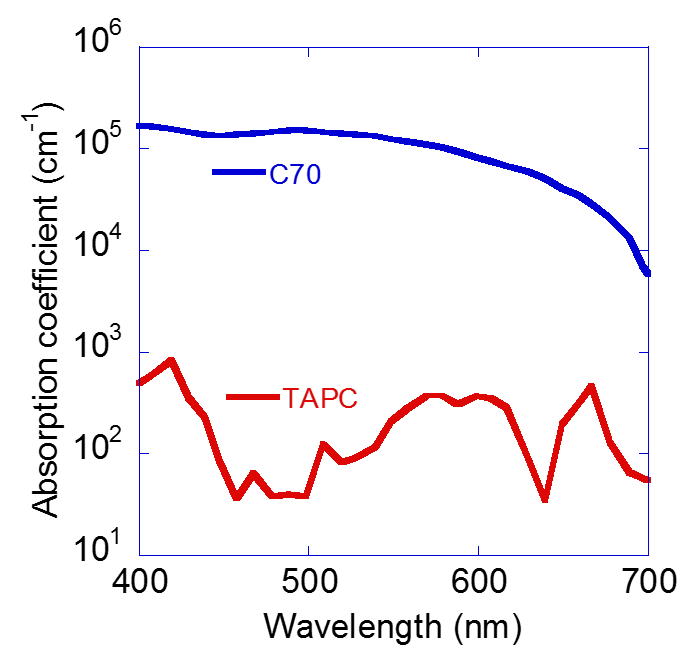


Figure 1. UV-Vis absorption spectra of thermally deposited C70 (blue) and TAPC (red) films on glass.


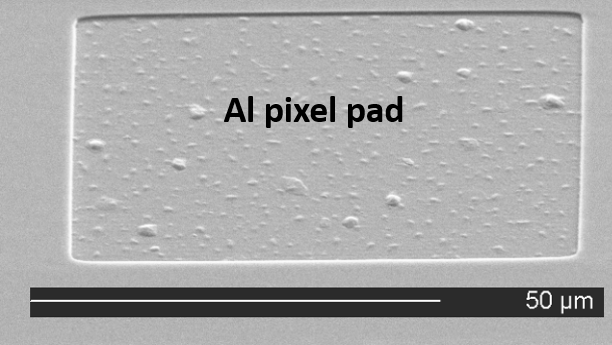


Figure 2. Top view SEM image showing surface roughness of aluminum pixel pads of the CMOS ROIC substrate.


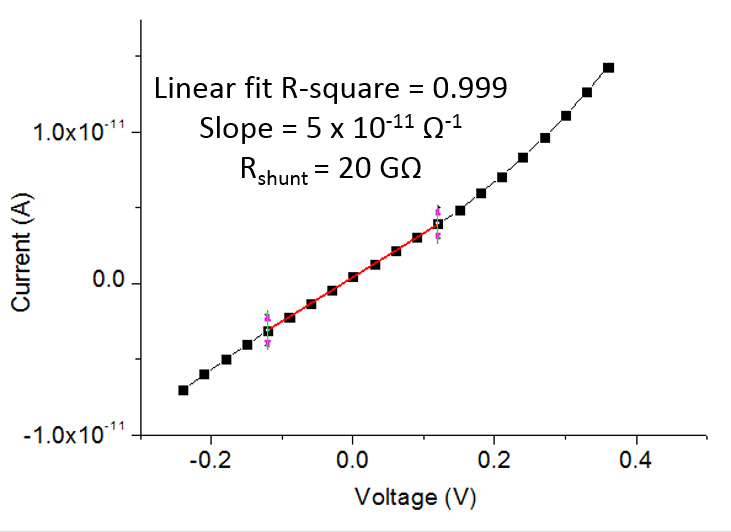


Figure 3. Linear fit (red line) of the dark current (black symbol) of device shown in Fig. 2a. Shunt resistance (R_shunt_) of the photodiode is approximated from the inverse of the slope of the curve at around V=0.


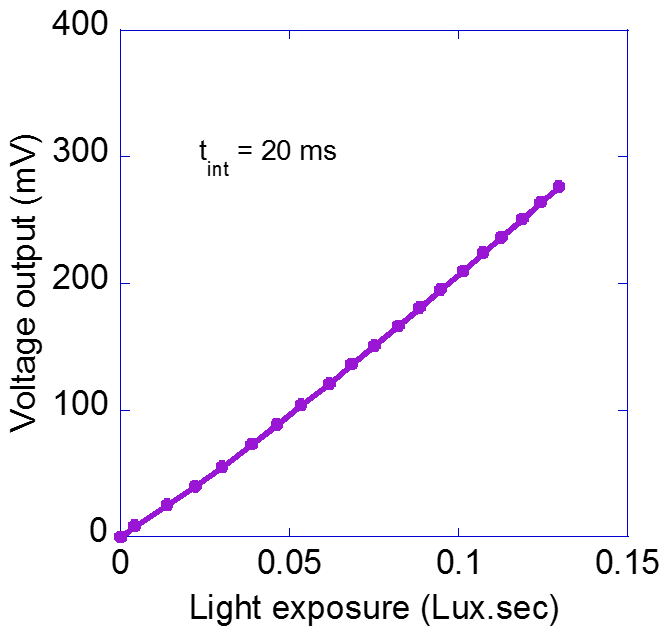


Figure 4. Pixel output response as a function of light exposure illuminated using a green light emitting diode of wavelength 523 nm. Pixel characterization was done under different light exposure by varying the light intensity from 5.36 x 10^-7^ mWcm^-2^ to 6.48 x 10^-3^ mWcm^-2^ at a 20 ms integration time. Pixel sensitivity calculated from the slope of the curve was 2.2 V/Lux.sec.

Specific detectivity (D^*^)

Specific detectivity (in unit- cm Hz^1/2^ W^-1^ or Jones) for a photodiode is given by

$D^{*}=\frac{R\sqrt{A\Delta f}}{i_{n}}$ (1)

where *R* is the responsivity (AW^-1^), *A* is the device area (cm^2^), $\Delta f$is the electrical bandwidth (Hz), and *i_n_* is the noise current (A).

Assuming flicker noise (1/f noise) and generation-recombination noises are negligible compared to shot noise and thermal noise, the noise current can be expressed as

*i_n_* = $\left[i_{shot}^{2}+i_{thermal}^{2}\right]^{1/2}$ = $\left[2qJ_{dark}\Delta f+\frac{4kT\Delta f}{R_{shunt}}\right]^{1/2}$ (2)

where *q* is the elementary charge, *J_dark_* is the photodiode dark current (A), *k* is the Boltzmann constant, *T* is the temperature, and *R_shun_*_t_ is the effective shunt resistance of the photodiode (Ω).

Taking the typical values of our device (Fig. 2a) *J_dark_* = 2.4 x 10^-10^ (A) at V = -1 V, *R_shun_*_t_ ̴ 20 GΩ (see Fig. 3, supplementary Information) the calculated shot noise current of 8.7 x 10^-15^ A was almost an order higher than the thermal noise current of 0.9 x 10^-15^ A for $\Delta f$= 1 Hz.

At 500 nm under -1 V the calculated detectivity (from equation (1)) was ̴ 6 x 10^12^ Jones.

Conversion gain (CG)

Conversion gain (V/e^-^) was calculated from the slope of the curve of voltage output (V) vs number of photogenerated electrons (*n_e_*). *n_e_* was calculated in the following way.

External quantum efficiency (EQE) of a photodiode is defined as the ratio of number of charge carriers collected at the electrode to the number of photons (*n_p_*) striking the photoactive area.

$EQE=\frac{n_{e}}{n_{p}}$ (3)

*n_p_* was calculated using below expression

$n_{p}=\frac{A\cdot E\cdot t_{\mathrm{int}}}{hc/\lambda}$ (4)

where A is the pixel size (cm^2^), E is the optical input power (Wcm^-2^), *t_int_* is the integration time (sec), h is the Planck’s constant (Joule.sec), c is the speed of light (cm/sec), and $\lambda$is the wavelength of light (523 nm) are known parameters.

From the calculated *n_p_* and taking EQE value of 25 % at 523 nm (Fig. 2b), *n_e_* was calculated using equation (2). Finally, output voltage was plotted as a function of *n_e_* whose slope gave the conversion gain of the hybrid pixel (Fig 6a).
